# Building sustainable capacity to adopt, adapt or develop child health guidelines, Malawi, Nigeria and South Africa

**DOI:** 10.2471/BLT.24.291564

**Published:** 2024-09-02

**Authors:** Tamara Kredo, Solange Durão, Emmanuel Effa, Celeste Naude, Michael McCaul, Amanda Brand, Simon Lewin, Claire Glenton, Susan Munabi-Babigumira, Elodie Besnier, Trudy D Leong, Bey-Marie Schmidt, Nyanyiwe Mbeye, Ameer Hohlfeld, Anke Rohwer, Tandekile Lubelwana Hafver, Nicolas Delvaux, Lungiswa Nkonki, Funeka Bango, Emma Thompson, Sara Cooper

**Affiliations:** a Health Systems Research Unit, South African Medical Research Council, Francie van Zijl Drive, Parow Valley, Cape Town, 7505, South Africa.; b Department of Medicine, Faculty of Clinical Sciences, University of Calabar, Cross River, Nigeria.; c Centre for Evidence-based Health Care, Department of Global Health, Faculty of Medicine and Health Sciences, Stellenbosch University, Cape Town, South Africa.; d Department of Health and Functioning, Western Norway University of Applied Sciences, Bergen, Norway.; e Department of Health Sciences in Ålesund, Norwegian University of Science and Technology, Ålesund, Norway.; f Department of Epidemiology and Biostatistics, Kamuzu University of Health Sciences, Blantyre, Malawi.; g MAGIC Evidence Ecosystem Foundation, Oslo, Norway.; h Division of Health System and Public Health, Department of Global Health, Faculty of Medicine and Health Sciences, Stellenbosch University, Cape Town, South Africa.; i Cochrane, London, England.; j Cochrane South Africa, South African Medical Research Council, Cape Town, South Africa.

## Abstract

**Problem:**

Many national child health guidelines in Malawi, Nigeria and South Africa are outdated and score poorly on rigorous methods and stakeholder participation.

**Approach:**

In line with the World Health Organization’s (WHO) emphasis on local guideline contextualization, the Global Evidence-Local Adaptation (GELA) project supported multistakeholder processes to adapt evidence-informed recommendations for child health in Malawi, Nigeria and South Africa. The GELA project team convened national steering groups, which conducted structured, iterative priority-setting exercises to identify priority topics. We identified appropriate source guidelines by systematically searching and screening available guidelines. We then matched recommendations in potential source guidelines to the relevant questions, and assessed the guidelines for timeliness and quality. Drawing on WHO’s guideline process, we applied the GRADE-ADOLOPMENT process to develop contextualized recommendations from existing guidelines. If no source guideline or reviews were identified, we conducted new evidence syntheses.

**Local setting:**

Malawi, Nigeria and South Africa are countries with varying health priorities and systems, all transitioning to universal health coverage. Guideline structures differ between countries, with processes largely led from national health ministries.

**Relevant changes:**

National guideline groups, supported by GELA researchers and government–academic partners, developed five contextually-tailored child health recommendations. For most of these recommendations, additional evidence was required to inform contextually appropriate national decision-making. Formal capacity-building and on-the-job learning enhanced the competencies of national contributors and researchers in evidence-informed decision-making.

**Lessons learnt:**

Developing context-relevant recommendations requires considerable resources and time. Further investment in strengthening local capacity is needed for sustainable national guideline development.

## Résumé

**Problème:**

En Afrique du Sud, au Malawi et au Nigeria, de nombreuses lignes directrices nationales relatives à la santé infantile sont obsolètes et affichent de piètres résultats en termes de rigueur des méthodes et d'implication des parties prenantes.

**Approche:**

S'alignant sur l'importance accordée par l'Organisation mondiale de la Santé (OMS) à la contextualisation des lignes directrices locales, le projet Global Evidence – Local Adaptation (GELA) a apporté son soutien à divers processus de collaboration multilatérale afin d'adapter des recommandations sur la santé infantile étayées par des faits en Afrique du Sud, au Malawi et au Nigeria. L'équipe du projet GELA a constitué des groupes de pilotage nationaux, chargés d'effectuer des exercices itératifs et structurés de définition des priorités afin de répertorier les principaux thèmes. Nous avons identifié des lignes directrices de référence en procédant à une recherche et une sélection parmi celles disponibles. Ensuite, nous avons comparé les recommandations de ces potentielles lignes directrices de référence avec les questions pertinentes, puis nous avons évalué leur qualité et leur actualité. En nous fondant sur le processus d'élaboration de lignes directrices de l'OMS, nous avons appliqué la méthodologie GRADE-ADOLOPMENT pour formuler des recommandations contextualisées à partir de lignes directrices existantes. Si aucune analyse ni ligne directrice de référence n'avait pu être dégagée, nous établissions de nouvelles synthèses de preuves.

**Environnement local:**

L'Afrique du Sud, le Malawi et le Nigeria sont des pays aux priorités et systèmes de santé variables, qui opèrent tous trois une transition vers une couverture sanitaire universelle. Les structures des lignes directrices diffèrent d'un pays à l'autre, les processus étant généralement menés par les Ministères nationaux de la Santé.

**Changements significatifs:**

Les groupes chargés d'élaborer des lignes directrices nationales, avec l'aide des chercheurs GELA et des partenaires gouvernementaux et universitaires, ont émis cinq recommandations adaptées au contexte en matière de santé infantile. Dans la plupart des cas, la prise de décisions nationales contextualisées nécessitait des preuves supplémentaires. Le programme de renforcement des capacités et l'apprentissage sur le terrain ont permis d'améliorer les compétences des chercheurs et contributeurs nationaux dans les processus décisionnels étayés par des faits.

**Leçons tirées:**

Mettre au point des recommandations propres à chaque contexte demande du temps et des ressources considérables. D'autres investissements dans le renforcement des capacités locales sont indispensables pour pérenniser le développement national de lignes directrices.

## Resumen

**Situación:**

Muchas directrices nacionales de salud infantil en Malawi, Nigeria y Sudáfrica están obsoletas y obtienen una puntuación baja respecto a los métodos rigurosos y la participación de las partes interesadas.

**Enfoque:**

En consonancia con el énfasis de la Organización Mundial de la Salud (OMS) en la contextualización local de las directrices, el proyecto Global Evidence-Local Adaptation (GELA) apoyó procesos multisectoriales para adaptar las recomendaciones basadas en la evidencia a la salud infantil en Malawi, Nigeria y Sudáfrica. El equipo del proyecto GELA convocó a grupos directivos nacionales, que realizaron ejercicios estructurados e iterativos de fijación de prioridades para identificar los temas prioritarios. Se identificaron las directrices fuente apropiadas mediante la búsqueda sistemática y el análisis de las directrices disponibles. A continuación, se compararon las recomendaciones de las posibles directrices fuente con las preguntas pertinentes y se evaluó la oportunidad y la calidad de las directrices. A partir del proceso de elaboración de directrices de la OMS, se aplicó el proceso GRADE-ADOLOPMENT para desarrollar recomendaciones contextualizadas partiendo de las directrices existentes. Si no se identificaba ninguna directriz o revisión fuente, se realizaban nuevas síntesis de la evidencia.

**Marco regional:**

Malawi, Nigeria y Sudáfrica son países con prioridades y sistemas sanitarios diversos, todos ellos en transición hacia la cobertura sanitaria universal. Las estructuras de las directrices difieren de un país a otro, y los procesos se dirigen en gran medida desde los ministerios de sanidad nacionales.

**Cambios importantes:**

Los grupos nacionales de directrices, apoyados por investigadores del GELA y socios gubernamentales y académicos, elaboraron cinco recomendaciones de salud infantil adaptadas a cada contexto. Para la mayoría de estas recomendaciones, se necesitaron pruebas adicionales para fundamentar una toma de decisiones nacional adecuada al contexto. La capacitación formal y el aprendizaje en el lugar de trabajo mejoraron las competencias de los colaboradores e investigadores nacionales en la toma de decisiones basada en evidencias.

**Lecciones aprendidas:**

La elaboración de recomendaciones adaptadas al contexto requiere recursos y tiempo considerables. Se necesita una mayor inversión en el fortalecimiento de la capacidad local para el desarrollo sostenible de directrices nacionales.

## ملخص

**المشكلة:**

إن العديد من المبادئ التوجيهية الوطنية لصحة الطفل في ملاوي ونيجيريا وجنوب أفريقيا، أصبحت عتيقة وتسجل درجات ضعيفة فيما يتعلق بالأساليب الصارمة، ومشاركة أصحاب المصلحة.

**الأسلوب:**

تماشياً مع تركيز منظمة الصحة العالمية (WHO) على صياغة المبادئ التوجيهية المحلية، فإن مشروع التكيف العالمي للأدلة المحلية (GELA) قد دعّم عمليات أصحاب المصلحة المتعددين الساعية لتكييف توصيات صحة الطفل المستندة إلى الأدلة في ملاوي، ونيجيريا، وجنوب أفريقيا. عقد فريق مشروع GELA مجموعات تنظيمية وطنية، أجرت تدريبات منظمة ومتكررة لوضع الأولويات، وذلك بهدف تحديد الموضوعات ذات الأولوية. لقد قمنا بتحديد المبادئ التوجيهية ذات المصدر المناسب من خلال البحث المنهجي وفحص المبادئ التوجيهية المتاحة. ثم قمنا بمطابقة التوصيات في المبادئ التوجيهية ذات المصدر المحتمل مع الأسئلة ذات الصلة، وقمنا بتقييم المبادئ التوجيهية ذات المصدر من حيث التوقيت والجودة. وبالاعتماد على إلى عملية المبادئ التوجيهية التابعة لمنظمة الصحة العالمية، قمنا بتطبيق عملية GRADE-ADOLOPMENT لتطوير التوصيات المُصاغة من المبادئ التوجيهية الحالية. إذا لم يتم تحديد أي مبدأ توجيهي أو مراجعات للمصدر، فقد أجرينا تجميعات أدلة جديدة.

**المواقع المحلية:**

تعد كل من ملاوي ونيجيريا وجنوب إفريقيا من دول جنوب الصحراء الكبرى بأفريقيا ذات أولويات وأنظمة الصحة المختلفة، وكلها في حالة انتقال إلى التغطية الصحية الشاملة. تختلف هياكل المبادئ التوجيهية بين الدول، حيث تتم قيادة العمليات بشكل كبير من جانب وزارات الصحة الوطنية.

**التغيّرات ذات الصلة:**

قامت مجموعات المبادئ التوجيهية الوطنية، بدعم من الباحثين لدى GELA والشركاء الأكاديميين الحكوميين، بتطوير خمس توصيات خاصة بصحة الطفل مصممة حسب الصياغة. بالنسبة لمعظم هذه التوصيات، كانت هناك حاجة إلى أدلة إضافية لتوعية عملية صنع القرار الوطني المناسبة للصياغة. إن كل من بناء القدرات الرسمية، والتعلم أثناء العمل، قد عززا من كفاءات المساهمين والباحثين الوطنيين في عملية صنع القرار المعتمدة على الأدلة.

**الدروس المستفادة:**

إن تطوير التوصيات المناسبة للصياغة يتطلب مواردًا ووقتًا كبيرين. هناك حاجة إلى مزيد من الاستثمار في تعزيز القدرات المحلية من أجل تطوير مبادئ توجيهية وطنية مستدامة.

## 摘要

**问题:**

马拉维、南非和尼日利亚的许多国家儿童健康指南已经过时，在严谨的方法和利益攸关方的参与度方面得分很低。

**方法:**

根据世界卫生组织 (WHO) 对当地指南情境化处理的重视，全球证据-地方调整 (GELA) 项目支持多方利益攸关方参与流程，以调整对马拉维、南非和尼日利亚儿童健康的循证建议。GELA 项目团队召集了各种国家指导小组，这些指导小组开展了结构化的、反复的优先事项设定练习活动，以确定优先事项主题。我们对现有指南进行了系统的检索和筛选，以确定合适的来源指南。然后，我们将潜在来源指南中的建议与相关问题进行匹配，并评估指南的及时性和质量。借鉴 WHO 的指南流程，我们应用了 GRADE-ADOLOPMENT 流程，基于现有指南制定了情境化处理建议。如未能确定来源指南或评论，我们则进行新的证据合成。

**当地状况:**

马拉维、南非和尼日利亚是撒哈拉以南的非洲国家，其卫生优先事项和体系各不相同，但都在向全民健康覆盖目标迈进。各国指南的框架结构各不相同，但流程主要由国家卫生部主导。

**相关变化:**

在 GELA 研究人员和政府学术合作伙伴的支持下，国家指南小组制定了五项针对具体情况的儿童健康建议。对于其中的大部分建议，均需要更多的证据来表明其可用于符合具体情况的国家的决策。正式的能力建设和在职学习提高了国家贡献者和研究人员在循证决策方面的能力。

**经验教训:**

制定与具体情况相关的建议需要耗费大量的资源和时间。需要增加投资以提升地方能力，从而制定可持续的国家指南。

## Резюме

**Проблема:**

Многие национальные руководящие принципы по охране здоровья детей в Малави, Нигерии и Южной Африке устарели и не соответствуют требованиям в отношении использования точных методов и участия заинтересованных сторон.

**Подход:**

В соответствии с акцентом Всемирной организации здравоохранения (ВОЗ) на адаптацию рекомендаций к местным условиям в рамках проекта «Глобальная доказательная база – местная адаптация» (Global Evidence-Local Adaptation, GELA) была оказана поддержка многосторонним процессам адаптации научно обоснованных рекомендаций по охране здоровья детей в Малави, Нигерии и Южной Африке. Командой проекта GELA были созваны национальные руководящие группы, которые провели структурированные, многократно повторяющиеся мероприятия по определению приоритетных тем. Для определения подходящих руководств был проведен систематический поиск и скрининг доступных руководящих принципов. Затем в потенциальных источниках были сопоставлены рекомендации по соответствующим вопросам и проведена оценка руководящих принципов на предмет своевременности и качества. На основании процесса разработки рекомендаций ВОЗ был применен процесс GRADE-ADOLOPMENT для разработки контекстуальных рекомендаций на основе существующих руководящих принципов. Если не было выявлено ни одного руководства или обзора, синтез доказательств проводился заново.

**Местные условия:**

Малави, Нигерия и Южная Африка – страны Африки к югу от Сахары с различными приоритетами и системами здравоохранения, которые переходят ко всеобщему охвату населения услугами здравоохранения. Структуры руководящих принципов в разных странах различны, и в основном процессами руководят национальные министерства здравоохранения.

**Осуществленные перемены:**

Национальными группами по разработке руководящих принципов при поддержке исследователей GELA и государственных академических партнеров были разработаны пять рекомендаций по охране здоровья детей с учетом конкретных условий. Для большинства этих рекомендаций требуются дополнительные доказательства для принятия решений на национальном уровне с учетом конкретных условий. Благодаря укреплению потенциала и обучению на рабочем месте повысилась компетентность национальных специалистов и исследователей в вопросах принятия решений на основе фактических данных.

**Выводы:**

Разработка учитывающих контекст рекомендаций требует привлечения значительных ресурсов и времени. Для устойчивой разработки национальных руководящих принципов необходимы дальнейшие инвестиции в укрепление местного потенциала.

## Introduction

Implementation of evidence-informed health guidelines tailored to the health system contexts of low- and middle-income countries is needed to improve health outcomes. Evidence-informed health guidelines are documents containing clinical, public health or health systems recommendations for optimizing health outcomes.^1^ These guidelines are essential for improving health-care quality, standardizing care, driving funding decisions and enhancing access to care.^1^ However, de novo guideline development is resource intensive. Therefore, adopting or adapting guidelines aims to avoid duplication of efforts and research waste by using available guidelines and systematic reviews instead of starting from scratch. However, guideline adoption may not be appropriate in all settings due to differences in health systems and local applicability of interventions.^2^^–^^5^ Adaptation considers local feasibility and affordability before introducing new interventions, which is important in overburdened health systems.

In practice, however, the methods of adopting and adapting guidelines, as well as the decision-making behind them, are poorly understood or reported across the World Health Organization (WHO) African Region. More evidence is needed on how guideline adaptation works, and the contexts and factors that affect guideline adaptation in varying settings within the region. There is also a growing appreciation for strengthening national evidence systems through capacity-building and shifting decision-making power to local levels.^6^

In 2022, we conducted a scoping review of national child health guidelines in Malawi, Nigeria and South Africa. We found challenges in accessing guidelines and critical gaps in covered topics. Many guidelines were outdated and scored poorly on rigorous methods and stakeholder participation. Additionally, many were adapted from global sources like WHO, without explicit mention.^7^

In response to poor child health outcomes and gaps in evidence-informed child health guidelines, the Global Evidence-Local Adaptation (GELA) project, established in 2022, explores and strengthens approaches for adopting or adapting available guidelines. The aim is to ensure transparent, credible, contextually-tailored guidance that can improve child health in Malawi, Nigeria and South Africa.^8^ GELA also aims to build sustainable in-country capacity around best practices for evidence-informed decision-making. The project builds on collaborations within the Cochrane Africa Network, and established relationships with global partners.

**Fig. 1:**
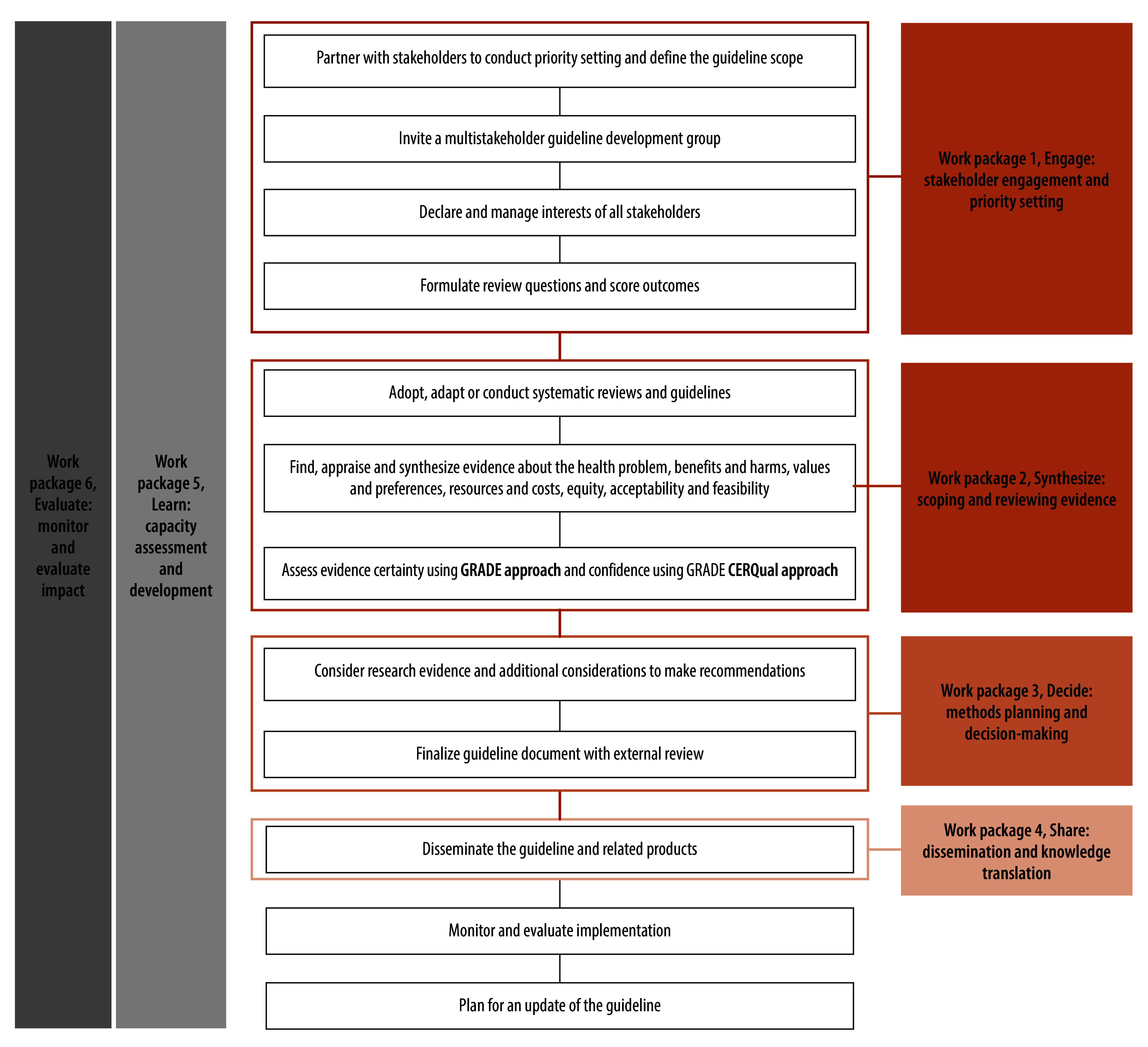
Project process to adopt, adapt or develop child health guidelines, Malawi, Nigeria and South Africa

Note: the process is aligned with the World Health Organization guideline development process.^1^

The overall approach of the GELA project aligns with the stepwise WHO guideline development process (Fig. 1).^1^ Here we describe our work with the first two work packages along with capacity development activities in 2022–2024.

## Local setting

Malawi, Nigeria and South Africa have diverse demographics, health burdens, gross domestic products and health systems arrangements. The countries’ governments have committed to achieving universal health coverage; however their child health outcomes still fall short of the sustainable development goal targets. 

The national health ministries, in collaboration with various professional groups and multilateral agencies, drive the adaptation or adoption of guidelines. These efforts are often funded externally by multilateral organizations or grants from international professional organizations. Despite these efforts, in Nigeria and Malawi, the lack of independent guideline standards, agencies or clearing-houses for appraising guidelines result in end-user-focused guidelines with low credibility regarding how the recommendations were reached. In contrast, the national health department in South Africa has a maturing guideline infrastructure, leading health technology assessment, medicine selection and national standard treatment guidelines. The use of global standards, such as the Grading of Recommendations Assessment, Development and Evaluation (GRADE) methods, is standard within the South African essential medicines list programme, but not yet widespread in the child health programme or related professional associations in the country.

## Approach

### Work package 1: engage

The GELA project team convened national steering groups with policy-makers, WHO country offices and civil society representatives in each country to work with GELA staff researchers, who were clinical epidemiologists, health economists, social scientists and communications officers. The team also invited representatives from the WHO Quality Assurance, Norms and Standards Department, the WHO Regional Office for Africa and other departments to participate in the GELA International Advisory Group.

National steering groups were responsible for identifying priority topics, suggesting guideline group members and providing advice to the GELA team. Between June 2022 and March 2023, the steering groups and GELA project team conducted structured, iterative priority-setting exercises, reviewing available national and global guidance, as well as identifying gaps in national practices.^9^ Each country’s steering group identified three priority questions.

The GELA team, in collaboration with the national ministries, convened multistakeholder guideline groups to develop evidence-informed recommendations for newborn and child health, aligning with both national and global standards for guideline development using GRADE methods. All contributors completed declarations of interest, which were reviewed for potential or actual conflicts of interest. Guideline groups provided further input to refine priority questions using the PICO format (population, intervention, comparator, outcomes), and to select and rate the importance of outcomes.

### Work package 2: synthesize

The overarching goal of this stage was to support the guideline group to make guideline judgements, by obtaining appropriate evidence for the evidence-to-decision tables, including evidence on benefits and harms, equity, feasibility, acceptability and resources. Instead of initiating new evidence reviews, we used GRADE-ADOLOPMENT, an evidence-to-decision framework-based approach to adopt, adapt or create contextualized recommendations from source guidelines and available evidence syntheses.^3^^,^^10^

The steps for each priority question are presented in Table 1. First, we identified appropriate source guideline(s) by systematically searching and screening available guidelines and creating a shortlist of potentially appropriate guideline(s). We then matched recommendation(s) in potential source guideline(s) to the PICO-formatted question, and assessed the guideline for timeliness, quality (using key Appraisal of Guidelines, Research and Evaluation version II (AGREE II) domains), use of GRADE methods and availability of evidence-to-decision tables. We judged a guideline appropriate if it had a well-matched recommendation to the PICO-formatted question, a clear pathway from an effectiveness systematic review to the recommendation, sufficient methodological quality and was up-to-date.

**Table 1:** Approaches and steps for evidence scoping and synthesis for developing, adopting or adapting available guidelines, Malawi, Nigeria, South Africa, 2022–2024

Evidence-to-decision criteria	Evidence category		
	Effectiveness (benefits and harms)	Costs and resources	Acceptability, feasibility, equity (qualitative evidence)
**Malawi: PICO question 1, early versus delayed enteral nutrition in critically ill children under 12 years**			
Appropriate source guideline?	No		
Source guideline evidence?	NA	NA	NA
Scoping of available syntheses?	No suitable systematic review identified	No suitable systematic review or economic evaluation identified	No suitable qualitative evidence synthesis found
What synthesis was done?	New systematic review conducted	New economic evaluation conducted	New qualitative evidence synthesis conducted
**Nigeria: PICO question 1, health worker-related interventions to improve compliance with hand hygiene recommendations for infection prevention and control in hospitalized neonates and infants**			
Appropriate source guideline?	No		
Source guideline evidence?	NA	NA	NA
Scoping of available syntheses?	No suitable systematic review identified	No suitable systematic review or economic evaluation identified	Qualitative evidence synthesis found, but low- and middle-income country evidence insufficient
What synthesis was done?	New systematic review conducted	New economic evaluation conducted	New supplemental targeted qualitative evidence synthesis conducted focusing on African settings
**Nigeria: PICO question 2, early versus delayed enteral feeding for improving outcomes in low-birthweight and preterm infants**			
Appropriate source guideline; AGREE II score,%^a^	*WHO recommendations for care of preterm or low-birthweight infants*^11^ Domain 1: 86%; Domain 3: 94%; Domain 6: 100%^a^		
Source guideline evidence?	Systematic review found	No suitable systematic review or economic evaluation identified	Two partially relevant qualitative evidence syntheses found
Scoping of available syntheses?	NA	No suitable systematic review or economic evaluation identified	NA
What synthesis was done?	None	New economic analysis conducted	Used related qualitative evidence synthesis prepared for Malawi question for equity considerations
**South Africa: PICO question 1, oral iron supplementation for anaemia prevention in children aged 6 to 23 months**			
Appropriate source guideline; AGREE II score,%^a^	*Guideline: daily iron supplementation in infants and children*^12^ Domain 1: 94%; Domain 3: 82%; Domain 6: 100%		
Source guideline evidence?	Systematic review found, out-of-date	No systematic review or economic evaluation	No qualitative evidence synthesis found
Scoping of available syntheses?	Identified recent appropriate systematic review with good methodological quality, but addressing wider population, 6–59-month-old children	No appropriate systematic review or economic evaluation	No appropriate qualitative evidence synthesis
What synthesis was done?	Partial update of appropriate systematic review, including only studies in 6–23-month-old children, updated search	New economic evaluation conducted	New qualitative evidence synthesis conducted
**South Africa: PICO question 2, family-centred support and post-discharge preparation interventions for families with preterm and low-birthweight infants**			
Appropriate source guideline; AGREE II score,%^a^	*Guideline: daily iron supplementation in infants and children*^12^ Domain 1: 94%; Domain 3: 82%; Domain 6: 100%		
Source guideline evidence?	Systematic review found, preprint low quality score	No systematic review or economic evaluation	Qualitative evidence synthesis found, with low- and middle-income country evidence missing
Scoping of available syntheses?	NA	Some partially relevant suitable systematic reviews or economic evaluations identified but not comprehensive or usable	NA
What synthesis was done?	Update of systematic review from source guideline	New economic analysis undertaken	New supplemental mini-qualitative evidence synthesis conducted with South African focus

ARGEE II: Appraisal of Guidelines, Research and Evaluation version II; NA: not applicable; PICO: Population, Intervention, Comparator, Outcome; WHO: World Health Organization

^a^ We only focused on domain 1: scope and purpose; domain 3: rigour of development; and domain 6: editorial independence of the AGREE II tool.

If we identified an appropriate source guideline, we assessed whether evidence underpinning the recommendation for the evidence-to-decision criteria was appropriate. This assessment involved judging whether the underlying evidence aligned with the PICO-formatted priority question and guideline context, was up-to-date and of sufficient quality (for example, moderate to high using the AMSTAR-2 appraisal tool). If available, we also assessed the evidence-to-decision table. We performed this step for each evidence category: effectiveness, resources, acceptability, equity and feasibility. We then decided on the most efficient pathway to obtain appropriate evidence for evidence-to-decision criteria for each evidence category, which was either (i) re-use the evidence from the source guideline; (ii) update the evidence; (iii) partially update the evidence; (iv) scope for appropriate available syntheses; or (v) conduct new reviews.

If we did not identify an appropriate source guideline for the priority PICO-formatted question or appropriate available syntheses, we conducted new reviews for each evidence category.

GELA researchers conducted and mentored others in conducting quantitative and qualitative evidence syntheses, while health economists supported national costing to inform evidence-to-decision tables.

There was substantial investment in capacity-building through workshops; accredited postgraduate university short courses; cross-country community of practice sessions for the steering group, guideline group and researchers in all countries to share learning; and informal experiential on-the-job learning for steering and guideline group members and GELA research staff (Table 2). 

**Table 2:** Capacity-building initiatives for adopting or adapting available guidelines, Malawi, Nigeria, South Africa, 2022–2025

Capacity-building activity	Target audience	Aim	Format
Primer in systematic reviews, short course	Guideline group and steering group members from Malawi, Nigeria and South Africa	To increase capacity to find, read and interpret systematic reviews of effects	8-week online course, including self-study and weekly Q&A sessions. Offered twice per year
WHO guideline simulation workshop	Guideline group and steering group committee members from all GELA partners	To increase capacity to participate in a guideline development group meeting	Half to full day, in-person workshop in respective countries. Offered once in each country
Bespoke training workshops^a^	GELA researchers from all GELA partners	To build capacity in various qualitative research skills	1–2 hour online training across countries. Offered as needed
Clinical practice guidelines short course at Stellenbosch University, South Africa	GELA researchers from all GELA partners	To increase capacity to understand the purpose of clinical practice guidelines, different approaches to developing guidelines, implementation strategies and monitoring and evaluation of evidence-based clinical practice guidelines	Semester-long, Master’s-level online course, comprising self-study, presentations, discussions and assessments. Offered once a year
GELA community of practice sessions	Guideline group and steering group committee members, and GELA researchers from all GELA partners	To enhance and share knowledge and context-relevant information by supporting interaction of peers across countries. Topics included: introduction to guidelines; GRADE and GRADE-CERQuaL approaches; equity considerations, and guideline adaptation methods	Instant messaging group for informal discussions, quarterly online meetings with structured topics and discussions
Learning-by-doing and experiential learning	GELA researchers and decision-makers from all GELA partners	To offer hands-on opportunities for individuals without experience in guideline and evidence synthesis methods to gain or enhance skills under the guidance of experienced professionals	Hands-on experience, on-the-job training and mentorship

CERQuaL: Confidence in the evidence from reviews of qualitative research; GELA: Global Evidence – Local Adaptation; GRADE: Grading of recommendations, assessment, development and evaluation; Q&A: questions and answers.

^a^ Including training in qualitative evidence synthesis, software-specific training, user-testing, observational research and the TRANSFER stakeholder engagement approach.

The review teams had numerous meetings to advance their syntheses, while steering or guideline groups had fewer meetings. In South Africa, most meetings were online, except for the guideline consensus meeting. In Malawi and Nigeria, guideline group members preferred in-person meetings. 

## Relevant changes

Despite the identification of high-quality guidelines, most priority questions required new or additional evidence to provide context-specific evidence for decision-making. 

Processes for identifying existing source guidelines, appraising available evidence, producing new evidence and completing evidence-to-decision tables took 7 to 12 months, depending on the complexity of the question, in-country and cross-country synthesis, methodologist capacities and project timelines.

Guideline groups met between October 2023 and March 2024, resulting in contextually relevant recommendations. Current work involves creating accessible guideline products with infographics, to be user-tested later in 2024. All processes have been systematically monitored, and an evaluation is underway using a longitudinal mixed-methods design.^13^

## Lessons learnt

To address gaps in the availability of context-relevant child health guidelines in Malawi, Nigeria and South Africa, the GELA project advanced national guideline decision-making aligning with WHO and GRADE guideline development approaches. Through this process, we supported government–academic partnerships in each country to identify child health priorities, gather global evidence from available guidelines and conduct new reviews where needed. Most child health guideline questions were found in source guidelines, often from WHO, but we found that all required new or additional systematic reviews, including of effectiveness and qualitative evidence. These reviews ensured that recommendations were context-relevant, based on the latest research and strengthened local capacity for evidence production. For example, our multicountry GELA teams conducted collaborative reviews of qualitative evidence, providing insights into factors affecting the acceptability and feasibility of interventions, informing implementation considerations and developing team members’ skills in this synthesis approach. Furthermore, our health economic analyses provided valuable evidence on context-relevant resource use and cost–effectiveness evidence. 

A key goal was to minimize duplication and devise resource-efficient, coordinated methods, and we made considerable efforts to achieve this goal. However, our experience suggests that developing child health recommendations in each country is still time- and human resource-intensive due to the need to address gaps in the global evidence and to ensure context relevance. We found that meeting global standards for guideline adaptation required investment in a range of skillsets at the national level, such as quantitative and qualitative systematic review methods, health economics, project management and knowledge translation. In addition, the project exposed a large number of government officials and academics working on child health to GRADE guideline methods. Over time, these investments should provide new guideline methodologists from the region, and enhance national capacity to adapt global guidelines and ensure relevance to the national context (Box 1).

Box 1 **Summary of main lessons learnt**

- Strengthening relationships among ministries, researchers and multilateral partners is important for successful national guideline work and for enhancing in-country capacity for evidence-informed decision-making.
- Developing fit-for-purpose child health guideline recommendations for each country requires considerable resources and time, as existing systematic review evidence may not always be readily usable or up-to-date and new evidence is often needed to support context-relevant guideline processes.
- Further investment in strengthening local capacity and embedding standardized processes are essential for sustaining the advances made in national capacity to adapt global guidelines.

Long-term investment in strengthening national decision-making capacity is crucial for the future sustainable development of evidence-informed decision-making in low- and middle-income countries.^5^ Strengthening decision-making and improving national guideline infrastructures would enhance the capability of guideline panels and technical teams to produce high-quality guidelines.
